# Prevalence and factors associated with syphilis among men who have sex with men in Brazil

**DOI:** 10.3389/fpubh.2025.1465799

**Published:** 2025-05-08

**Authors:** Paulo Roberto Queiroz, Marquiony Marques dos Santos, Ana Karla Bezerra Lopes, Jose Adailton da Silva, Brunno Giordano da Silva Aranha Filho, Thalia Giesta Costa, André Carlos Nogueira Bezerra, Kenio Costa Lima

**Affiliations:** ^1^Departamento de Ciências da Saúde, Programa de Pós-graduação em Ciências da Saúde, Universidade Federal do Rio Grande do Norte, Natal, Brazil; ^2^Departamento de Enfermagem, Universidade do Estado do Rio Grande do Norte, Caicó, Brazil; ^3^Departamento de Saúde Coletiva, Universidade Federal do Rio Grande do Norte, Natal, Brazil; ^4^Departamento de Medicina, Faculdade de Medicina, Universidade Federal do Rio Grande do Norte, Natal, Brazil

**Keywords:** MSM, mobile apps, syphilis, STIs, sexual behavior

## Abstract

**Purpose:**

This study aimed to analyze the prevalence of syphilis and assess the factors associated with its diagnosis throughout life.

**Methods:**

Cross-sectional study conducted from January to April 2022 with 812 MSM users of dating apps in Brazil. Sociodemographic, behavioral, and sexual aspects, health issues, and the use of dating apps variables were analyzed.

**Results:**

Multivariate logistic regression estimated the predictors of syphilis at a 95% confidence interval. The prevalence of syphilis diagnoses was 34.23% (95% CI = 30.94–37.46), and the factors associated with the diagnosis throughout life were age ≥ 30 years (adjusted prevalence ratio [aPR] = 1.49; adjusted 95% confidence interval [a95%CI] = 1.15–1.92), more than three sexual relations with Cis men (aPR = 1.40; a95%CI = 1.03–1.90), sex simultaneously with two people or more during social distancing (aPR = 1.31; a95%CI = 1.00–1.73), use of a licit (or illicit) substance in the last 3 months (aPR = 1.35; a95%CI = 1.05–1.74), using condom less than half of the time in anal sex in the last 3 months (aPR = 1.42, a95%CI = 1.07–1.89), being a PrEP user (aPR = 1.10; a95%CI = 0.81–1.51) and having used the Grindr® app in the last 3 months (aPR = 1.38; a95%CI = 1.04–1.83).

**Conclusion:**

The prevalence of syphilis among MSM users of dating apps is at worrying levels. Thus, frequent testing (less than 3 months for MSM using PrEP and semi-annual for everyone, regardless of the risk), access to DoxiPEP for this population, consistent use of condoms, and the use of apps as a strategic intervention and educommunication platforms are essential to prevent the spread of syphilis.

## 1 Introduction

The number of men who have sex with men (MSM) presenting syphilis infection is 15-fold higher than the general population worldwide (7.5% vs. 0.5%, respectively) (1). Syphilis is a sexually transmitted infection (STI) caused by *Treponema pallidum* that is preventable and curable (2). This infection affects the genitourinary system (3) and may impair the neurological, cardiovascular, and ocular systems (4), facilitating HIV transmission and acquisition (5).

Due to the impact of syphilis as a public health problem, the World Health Organization (WHO) launched a campaign in 2017 aiming to eliminate syphilis by 2030 by reducing new infections by 90% (6). Despite this campaign, the prevalence of syphilis infection is continuously increasing among MSM (1). Brazil has implemented measures under the supervision of the Ministry of Health (e.g., early treatment and mass testing) to reduce the new cases of syphilis (7, 8).

The factors that explain the prevalence of syphilis among MSM are the increased condomless anal sex among PrEP (Pre-Exposure Prophylaxis) users for HIV (9), substance abuse (10), and multiple sexual partners (11). In the United States and Western Europe, the resurgence of syphilis among MSM has been attributed to the expansion of sexual networks facilitated by dating apps (12).

The prevalence of syphilis was associated with the use of dating apps among MSM (12). However, this association has not yet been studied in Brazilian MSM (13–15). Thus, this study aimed to verify the prevalence of syphilis among Brazilian MSM users of dating apps and the factors associated with the diagnosis of syphilis throughout life, analyzing sociodemographic, behavioral, and sexual aspects, health issues, and use of apps to improve prevention, treatment, and help to achieve the national targets for incidence reduction of syphilis proposed by WHO and the country.

## 2 Materials and methods

### 2.1 Participant and recruitment

The cross-sectional study was conducted online, considering the five regions of Brazil (North, Northeast, South, Southeast, and Midwest) from January to April 2022.

The sample size calculation for infinite populations considered a prevalence of 50%, an absolute margin of error of 5%, a non-response rate of 20%, and an estimated proportion of the event of 50%. The calculation resulted in a sample size of 480 individuals. Since the study aimed to verify the association of some variables with the outcome, the sample size increased by 70%, totaling 816 individuals.

The allocation of individuals occurred by intentional sampling, using invitations sent via dating apps.

For recruitment, the researchers used boosting on the dating apps Grindr®, Scruff®, Hornet®, and Tinder® to target the MSM aged ≥ 18 years (age limit imposed by the apps). Using the direct chat with online users, the researchers sent an electronic link with the informed consent form and the questionnaire (Google Forms®).

Inclusion criteria considered MSM aged ≥ 18 years who used the dating apps at least once in the last month. Those who were foreigners were excluded.

### 2.2 Questionnaire

A form was created and validated (face-content) by three experts from the country, and was hosted on a data collection website that only allowed one response per internet protocol (IP) for security reasons. The form comprised 56 questions divided into four sections; most were multiple choice, and some of them were mandatory:

1. Sociodemographic (gender identity, sexual orientation, race or skin color, age group, educational level, work, occupation, income, region of residence, marital status, religiosity, religion, and whom you reside with);
2. Behavioral and sexual (impact of social distancing on sexual life, assessed using two categories: high or medium impact and low or none impact, practice of sex in the last 3 months, type of sexual partnership that had sex with, number of cis men who had sex with, practice of oral sex, number of oral sex with cis men in the last 3 months, type of oral sex in the last 3 months, frequency of condom use in oral sex, accepted money in exchange for sex, practice of paid sex in the last 3 months, considers to be a sex worker, practice of chemosex, practice of chemosex in the last 6 months, practice of chemosex in the last 2 years, sexual positioning, type of sexual partnership, fixed sexual partnership, casual sex during social isolation, sex with two or more people simultaneously during social isolation, use of licit or illicit substance in the last 3 months, substances used, frequency of condom use in anal sex in the last 3 months, and type of sex without a condom in the last 3 months);
3. Health issues (syphilis testing throughout life, positive test for syphilis, diagnosis of STIs throughout life, diagnosis of lifelong STIs, diagnosis of STIs in the last 6 months, STIs diagnosed in the last 6 months, use of PrEP, the start of PrEP use, unprotected sex with people positive for syphilis in the last 6 months, consumption of five or more doses of alcohol during about 2 hours in the last 3 months, access to public health service, last access to public health service, access to the Family Health Strategy/Community Health Agent, and have a supplementary health plan);
4. Use of the app (app as an environment where you met your partner, when they began using the app, frequency of app use, period of highest app use, week when most use the app, most used app in the last 3 months, and purpose of app usage).

### 2.3 Measures

The dependent variable was the positive test for syphilis if the individual was diagnosed at some point in time their life, categorized as yes or no; the “yes” answer was considered a diagnosis of syphilis. Thus, this variable was recategorized as “lifelong diagnosis of syphilis” for a better understanding of the research question and the results. Moreover, the other variables collected in the form were considered as covariates.

### 2.4 Statistical analysis

Data were tabulated and distributed in absolute numbers and percentages. Numerical variables were categorized by medians or tertiles based on a theoretical basis. Then, a Chi-square analysis identified the association between lifelong diagnosis of syphilis and sociodemographic, behavioral, and sexual aspects, health issues, and app use. The prevalence ratio (PR) was presented using a statistical significance of 95%.

A logistic regression model was performed using the stepwise forward method for identifying the independent predictors of lifelong diagnosis of syphilis; variables presenting a *p*-value < 0.200 were included. The order of variable entries in the model respected the theoretical and statistical model, as well as the absence of multicollinearity. Last, a residue analysis identified cases that could negatively influence the model. The adjusted prevalence ratio (aPR) and the adjusted 95% confidence interval (a95%CI) were calculated from logistic regression estimators; statistical significance was set at *p* < 0.05. All data were analyzed using SPSS (version 22.0).

### 2.5 Ethics

The study was approved by the research ethics committee of the Onofre Lopes University Hospital - HUOL of the Federal University of Rio Grande do Norte - UFRN in Natal - RN (no. 51618521.8.0000.5292).

## 3 Results

### 3.1 Sociodemographic aspects

A total of 818 MSM were recruited; four were excluded because they were foreigners, and two because they were under 18 years of age. Thus, 812 MSM were included in the analysis. Most users were male cis (92.7%), homosexual or gay (81.2%), white (52.0%), single (91.1%), and aged below 30 years (57.1%). In addition, 81.4% had at least higher education, 75.4% worked, and 33.4% earned between one and three minimum wages; 30.7% did not know how to answer in relation to occupation. About 28.1% lived in the Northeast region of Brazil, 36.8% lived alone, 48.6% had no religion, and those who had were mostly catholic (18.2%) (Supplementary Table 1).

### 3.2 Behavioral and sexual aspects

Regarding behavioral and sexual aspects, 69.6% of the users reported a high or medium impact of social distancing on sexual life, 85.2% had sex in 3 months, 81.7% reported more common sexual partnership with cis men, 42.7% with more than three partners, and 88.9% reported having oral sex. Considering the last 3 months, 49.3% reported oral sex with > 3 cis men, 74.1% related the type of relationship (receptive or insertive), 72.8% never used condoms in oral sex, and 80.9% did not accept money in exchange for sex. Despite this, 81.5% did not know or did not answer if they had paid for sex, and 82.5% did not know about relations with sex workers. In addition, most 68.1% did not perform chemosex practice, and 71.6% did not know or did not respond to the frequency of chemosex practice in the last 6 months or the last 2 years (72.2%). About 57.1% declared themselves versatile, 51.6% had casual sexual partnerships, 83.5% had casual sex during social distancing, but 54.9% denied simultaneous sex with two or more people during social distancing. Last, 52.0% denied using a licit or illicit substance in the last 3 months, but the most common substance was marijuana for those who used it (24.3%); most (55.9%) did not know or did not answer the question. Most (47.6%) used condoms in more than half of anal sex in the last 3 months, and 40.3% practiced the insertive role without a condom in the last 3 months (Supplementary Table 1).

### 3.3 Health issues and app usage

Regarding health issues, most (84.4%) were tested for syphilis throughout their lives: 50.1% tested negative, and 54.4% had already been diagnosed with STIs throughout their lives. In addition, 81.9% did not use PrEP, 85.2% did not know or did not respond to the beginning of its use, and 43.1% did not know whether the partner had a syphilis infection. Moreover, most (67.1%) drank five or more doses of alcohol within 2 hours in the last 3 months. Most (94.7%) had access to public health services; 43.1% occurred in the last month; 55.5% had access to the Family Health Strategy/Community Health Agent, and 54.1% had supplementary health insurance. Considering the use of the app, 61.7% met their partner in the app, 72.2% started using the app before the pandemic, 53% used it every day, 44.5% usually throughout the day, and 52.1% during the weekdays. The most used app by users in the last 3 months was Grindr® (59.4%), and the purpose of use was sex for 42.7% (Supplementary Table 1).

### 3.4 Prevalence of syphilis and factors associated with lifelong diagnosis of syphilis

The prevalence of lifelong diagnosis of syphilis among Brazilian MSM users of dating apps was 34.23% (95%CI = 30.94–37.46) in 2022 (Supplementary Table 1). The factors associated with this variable include age of 30 years or more (aPR = 1.49; a95%CI = 1.15–1.92), more than three sexual relations with cis men (aPR = 1.40; a95%CI = 1.03–1.90), sex simultaneously with two people or more during social distancing (aPR = 1.31; a95%CI = 1.00–1.73), use of licit or illicit substance in the last 3 months (aPR = 1.35; a95%CI = 1.05–1.74), frequency of condom use in anal sex in the last 3 months less than half of the time (aPR = 1.42, a95%CI = 1.07–1.89), being a PrEP user (aPR = 1.10; a95%CI = 0.81–1.51) and having used the Grindr® app in the last 3 months (aPR = 1.38; a95%CI = 1.04–1.83) (Supplementary Table 2).

## 4 Discussion

The prevalence of lifelong diagnosis of syphilis is at worrying levels. Factors associated with this diagnosis include the age of 30 years or older, more than three sexual relations with cis men, sex simultaneously with two people or more during social distancing, use of licit or illicit substance in the last 3 months, frequency of condom use in anal sex in the last 3 months in less than half of the time, being a PrEP user, and having used the Grindr® app in the last 3 months.

In the present study, the prevalence of syphilis among Brazilian men who have sex with men (MSM) users of dating apps was 34.23%, representing an increase of about 10,273% compared with the previous estimate of 0.33% (16). This variation may be related to the sample size; while our study focused exclusively on MSM who use dating apps, the other covered MSM in general. This increase indicates an ongoing epidemic with no signs of slowing down. Also, an effective strategy requires up-to-date information on the prevalence of syphilis among MSM to eliminate this disease as a public health threat (1, 2).

Although syphilis is treatable and curable (2), its prevalence rate among MSM in Latin America and the Caribbean exceeds 10.0% (1), and it is strongly associated with HIV infection and high-risk sexual behaviors (4). In addition, this population often remains invisible to government authorities, hampering access to public health services (17). The COVID-19 pandemic also contributed to the difficulties in accessing tests and treatments for STIs, which may have hindered the diagnosis and treatment of syphilis, increasing its prevalence (18).

This study identified a significant association between age group ≥ 30 years and lifelong diagnosis of syphilis. Older individuals present increased exposure time to *Treponema pallidum* throughout their lives, increasing the chances of developing the disease. Mendez-Lopez et al. found that older individuals living in Europe were more likely to be diagnosed with syphilis (19), corroborating our findings.

A significant association was also observed between the lifelong diagnosis of syphilis and the practice of simultaneous sexual activity with two or more people; the diagnosis was also significantly associated with the practice of more than three sexual relations during social distancing. These findings evidence that group sexual activities were maintained in this period. Thus, MSM requires more attention regarding health care, given the possibility of a higher risk for transmission of unrestrained infections.

An online study with 2,311 MSM from Brazil and Portugal showed that simultaneous relationships with more than one person trigger the low perception of risk for STIs because the previous history of their sexual partnerships, involvement of ineffective prevention methods, and greater use of drugs are not known (20).

In addition, the COVID-19 pandemic increased the risks of illness for the entire Brazilian population. Moreover, MSM were double exposed because they could be infected with syphilis or SARS-CoV-2 when looking for sexual partners presenting different levels of exposure to the virus (21).

In addition, the affective needs promoted by social distancing may have induced MSM to break isolation and seek sexual partners (18). This hypothesis is corroborated by the multivariate analysis, which evidenced the high impact of social distancing in the search for casual sexual partners (22). Thus, the findings of the present study corroborate other studies developed worldwide (23).

The insertion and good adherence to PrEP may also have influenced the greater practice of group sex during social distancing; however, the functionality of this drug was misunderstood. Some MSM believed that PrEP would prevent HIV and COVID-19. A multicenter study revealed that 21.8% of MSM used PrEP as a protective measure against SARS-CoV-2.

The composition of PrEP may have contributed to the erroneous perception of MSM. Therefore, MSM must be informed about the risks of group sex and the forms of prevention during their sexual activities; the COVID-19 prevention measures must also be clarified, highlighting vaccination as the main strategy.

In the present study, the use of licit or illicit substances in the last 3 months was associated with the lifelong diagnosis of syphilis, corroborating a previous study that identified a relationship between the use of illicit drugs and syphilis infection and other STIs in MSM (1, 10, 24).

MSM who abuse substances are more likely to seek out sexual partners using dating apps and engage in unprotected anal sex and risky sexual behaviors for syphilis transmission (4, 10, 11). Therefore, the substance use by this group may contribute to the spread of syphilis within this population.

In the present study, condom use in anal sex in the last 3 months less than half of the time was independently associated with the lifelong diagnosis of syphilis. The condom is a low-cost item that is easy to handle and store; however, it is still underused in anal sex, making behavioral patterns a risk factor for syphilis infection (11). Also, the infection risk during anal sex increases with each additional non-stable partner (19).

Thus, the increased condomless anal relations among MSM in recent years (25) reflects a change in the behavior pattern within this group. This population may present an increased confidence in antiretroviral treatment since it makes the individual undetectable and untransmittable when under HIV treatment. This reliability may influence their perceptions about the risk for other STIs (including syphilis) and lower their adherence to condoms.

Additionally, the use of PrEP may also be influencing the maintenance of risky behavior in sexual relations serodiscordant for HIV (26). The present study may justify this finding since PrEP use was significantly associated with the lifelong diagnosis of syphilis, corroborating other studies. Ayerdi Aguirrebengoa found that Spanish PrEP users had less condom sex and more bacterial STIs (9).

Despite the high efficacy of PrEP against HIV and its reduced incidence, the behavioral changes among PrEP users are concerning since the bacterial STIs increased after drug adherence. In this sense, group, casual, and condomless sex during anal sex may be part of the behavioral changes caused by adherence to PrEP (27). This identification corroborates other studies worldwide, which suggest a degree of risk compensation among individuals after starting PrEP (4, 27, 28).

Condom use in less than half of the anal sex performed in the last 3 months and PrREP use were associated with a lifelong diagnosis of syphilis. However, this study could not determine whether individuals using PrEP already had a higher frequency of condomless anal sex, and whether the PrEP use increased the number of condomless anal sex. Therefore, further studies must investigate the behavior changes after the initiation of PrEP use and its possible association with the increased incidence of syphilis.

Therefore, new strategies are needed to prevent and combat syphilis, as cases continue to increase despite quarterly screening performed during routine PrEP consultations. One of the main strategies is to perform biannual testing for all MSM, regardless of risk level. Moreover, DoxiPEP (a prophylactic antibiotic against STIs) must be included within the national health protocol. Individuals who use Doxycycline 200 mg orally in a single dose within 72 h after condomless sex are less likely to have syphilis (29).

Reducing the duration of the infectious period also could help syphilis control because identifying the primary lesions in places of difficult visualization (e.g., the vaginal or inside the anal canal) breaks the transmission chain of the disease. An Australian study reported that MSM who engaged in receptive anal sex were four-fold more likely to have secondary syphilis than those who engaged in insertive anal sex, suggesting that primary anorectal lesions are often lost (30). Thus, anal self-examination may be useful to identify syphilis lesions in the primary stage. Also, instructions on the risk of syphilis and the recognition of symptoms among MSM taking PrEP may strengthen preventive measures for syphilis infection.

Although the multivariate analysis encompassed three dating apps, the present study evidenced that MSM who predominantly used the Grindr® app in the last 3 months were significantly associated with lifelong diagnoses of syphilis.

Geolocation apps specializing in sexual encounters, such as Grindr®, allow users to create personalized profiles, chat, share photos, and their exact locations (31). In addition, the functionality of identifying close users simplifies the search for potential sexual partners (32).

In this context, the use of dating applications among MSM has been responsible for increasing risky sexual behaviors, raising STI rates (33), increasing the number of casual sexual partners, unprotected anal sex, and ignorance of the serological status of their last sexual partner (34).

All of these factors may explain in part the correlation between frequent Grindr usage in the last 3 months and lifelong diagnosis of syphilis. However, it is important to emphasize that this association does not imply causation, and further studies are needed to better understand this relationship.

When understanding Grindr® use is related to the diagnosis of syphilis, researchers and health managers need to collaborate with app developers to direct these technologies in the prevention and control of syphilis and other STIs. Dating applications can reach MSM, which is often difficult to access via other communication strategies (35).

## 5 Strengths and limitations

The present study used the same methods to assess self-reported STI diagnoses in all five regions of the country, focusing on the voluntary self-identification of MSM. However, the use of self-reported diagnoses may have limited the analysis. For instance, Information is limited on what testing methods were used to diagnose infections and what definitions were used for active syphilis. If syndromic approaches were used, we do not know whether and what type of diagnosis was communicated to our sample; that is, the proportion of syphilis diagnoses that were active infections requiring treatment is unknown because the information on subsequent STI treatment was not collected. In addition, self-reported data on diagnoses and behaviors may be subject to biases of social convenience (i.e., the use of illicit drugs), memory, and misattribution of symptoms to certain infections, as well as confusion between different STIs.

## 6 Conclusion

The prevalence of syphilis among MSM users of dating apps is at worrying levels, indicating that this disease is an ongoing epidemic with no signs of slowing down. Thus, frequent testing (less than 3 months for MSM using PrEP and semi-annual for everyone, regardless of the risk), access to DoxiPEP for this population, consistent use of condoms, and the use of apps as a strategic intervention and educommunication platforms are essential to prevent the spread of syphilis.

## Data Availability

The original contributions presented in the study are included in the article/Supplementary material, further inquiries can be directed to the corresponding author.
