# Supplementary material for: Prevalence and factors associated with syphilis among men who have sex with men in Brazil
Source: Front Public Health. 2025 May 8;13:1465799. doi: 10.3389/fpubh.2025.1465799 (PMC12094950; doi:10.3389/fpubh.2025.1465799)
Supplement: Supplementary file 1 [file Table_1.docx]

***Supplementary Material***

# Supplementary Tables

**Supplementary Table 1.** **Sociodemographic, behavioral, and sexual characteristics, health issues, and application use among MSM in Brazil (*N* = 812).**

|  | n | % |
| --- | --- | --- |
| **Sociodemographic characteristics** | | |
| Gender identity |  |  |
| Cis man | 753 | 92.7 |
| Transgender man | 15 | 1.8 |
| Non-binary | 24 | 3.0 |
| Do not know or do not answer | 20 | 2.5 |
| Sexual Orientation |  |  |
| Heterosexual | 4 | 0.5 |
| Homosexual or Gay | 659 | 81.2 |
| Bisexual | 137 | 16.9 |
| Do not know or Do not answer | 12 | 1.5 |
| Race or skin color |  |  |
| White | 422 | 52.0 |
| Black | 368 | 45.3 |
| Asian | 13 | 1.6 |
| Indigenous | 8 | 1.0 |
| Do not know/Do not answer | 1 | 0.1 |
| Age group |  |  |
| < 20 | 28 | 3.4 |
| 20 - 29 years old | 436 | 53.7 |
| 30 - 39 years old | 227 | 28.0 |
| 40 - 49 years old | 88 | 10.8 |
| 50 - 59 years old | 31 | 3.8 |
| Do not know or do not answer | 2 | 0.2 |
| Educational level |  |  |
| Elementary school | 2 | 0.2 |
| High school | 147 | 18.1 |
| Higher education | 406 | 50.0 |
| Post graduate | 255 | 31.4 |
| Do not know or do not answer | 2 | 0.2 |
| Works |  |  |
| No | 199 | 24.5 |
| Yes | 612 | 75.4 |
| Do not know or do not answer | 1 | 0.1 |
| Occupation |  |  |
| Student | 169 | 20.8 |
| Employed freelance professional | 100 | 12.3 |
| Self-employed professional | 117 | 14.4 |
| Employee or office worker | 11 | 1.4 |
| Do not know or Do not answer | 249 | 30.7 |

**Table 1 (continuation)**

|  | n | % |
| --- | --- | --- |
| Income |  |  |
| One MW or less | 107 | 13.2 |
| Between one and three MW | 271 | 33.4 |
| Three MW or more and less than five MW | 182 | 22.4 |
| Five or more MW | 239 | 29.4 |
| Do not knowor do not answer | 13 | 1.6 |
| Region of residence |  |  |
| North | 97 | 11.9 |
| Northeast | 228 | 28.1 |
| Midwest | 131 | 16.1 |
| Southeast | 216 | 26.6 |
| South | 140 | 17.2 |
| Marital status |  |  |
| Single | 740 | 91.1 |
| Not single | 72 | 8.9 |
| Religiosity |  |  |
| No | 395 | 48.6 |
| Yes | 388 | 47.8 |
| Do not know or do not answer | 29 | 3.6 |
| Religion |  |  |
| No religion | 395 | 48.6 |
| Catholicism | 148 | 18.2 |
| Spiritist | 68 | 8.4 |
| Protestant or evangelical | 54 | 6.7 |
| Religion of African origin | 66 | 8.1 |
| Other (Jewish, Buddhist, among others) | 42 | 5.1 |
| Do not knowor do not answer | 39 | 4.8 |
| Whom resides with |  |  |
| Alone | 299 | 36.8 |
| With parents | 246 | 30.3 |
| With relatives | 128 | 15.8 |
| With colleague or friend or partner | 138 | 17.0 |
| Do not know or do not answer | 1 | 0.1 |
| **Behavioral and sexual aspects** | | |
| Impact of social isolation on sex life |  |  |
| High impact | 303 | 37.3 |
| Medium impact | 262 | 32.3 |
| Low impact | 188 | 23.2 |
| None | 54 | 6.7 |
| Do not know/Do not answer | 5 | 0.6 |

**Table 1 (continuation)**

|  | n | % |
| --- | --- | --- |
| Sex in the last three months |  |  |
| Yes | 692 | 85.2 |
| No | 120 | 14.8 |
| Sexual partnership |  |  |
| Transgender men | 2 | 0.2 |
| Cis men | 663 | 81.7 |
| Cis and trans man | 11 | 1.4 |
| Do not know or do not answer | 16 | 2.0 |
| Number of cis men who had sex with |  |  |
| Up to three cis men | 308 | 37.9 |
| More than three cis men | 347 | 42.7 |
| Do not know or do not answer | 157 | 19.3 |
| Oral sex |  |  |
| Yes | 722 | 88.9 |
| No | 87 | 10.7 |
| Do not know or do not answer | 3 | 0.4 |
| Number of oral sex with cis men in the last three months |  |  |
| Up to three cis men | 372 | 45.8 |
| More than three cis men | 400 | 49.3 |
| Do not know or do not answer | 40 | 4.9 |
| Sex in the last three months |  |  |
| Receptive and insertive | 602 | 74.1 |
| Insertive | 65 | 8.0 |
| Receptive | 50 | 6.2 |
| Do not know or do not answer | 95 | 11.7 |
| Frequency of condom use in oral sex |  |  |
| None | 591 | 72.8 |
| Some or all the times | 125 | 15.4 |
| Do not know or do not answer | 96 | 11.8 |
| Accepted money in exchange for sex |  |  |
| Yes | 152 | 18.7 |
| No | 657 | 80.9 |
| Do not know or do not answer | 3 | 0.4 |
| Paid for sex in the last three months |  |  |
| Yes | 32 | 3.9 |
| No | 118 | 14.5 |
| Do not know or do not answer | 662 | 81.5 |
| Sex worker |  |  |
| Yes | 12 | 1.5 |
| No | 130 | 16.0 |
| Do not know or do not answer | 670 | 82.5 |

**Table 1 (continuation)**

|  | n | % |
| --- | --- | --- |
| Chemosex practice |  |  |
| Yes | 250 | 30.8 |
| No | 553 | 68.1 |
| Do not know or do not answer | 9 | 1.1 |
| Chemosex practice in the last six months |  |  |
| Up to three times | 156 | 19.2 |
| More than three times | 75 | 9.2 |
| Do not know or do not answer | 581 | 71.6 |
| Chemosex practice in the last two years |  |  |
| Up to three times times | 125 | 15.4 |
| More than three times | 101 | 12.4 |
| Do not know or do not answer | 586 | 72.2 |
| Sexual Positioning |  |  |
| Versatile | 464 | 57.1 |
| Insertive anal only | 151 | 18.6 |
| Receptive anal only | 155 | 19.1 |
| Gouines | 25 | 3.1 |
| Do not know or do not answer | 17 | 2.1 |
| Sexual partnership |  |  |
| Casual | 419 | 51.6 |
| Fixed | 72 | 8.9 |
| Casual or fixed partner | 290 | 35.7 |
| Do not know or do not answer | 31 | 3.8 |
| Casual sex during social isolation |  |  |
| Yes | 678 | 83.5 |
| No | 131 | 16.1 |
| Do not know or do not answer | 3 | 0.4 |
| Sex with two people or more simultaneously during social isolation |  |  |
| Yes | 360 | 44.3 |
| No | 446 | 54.9 |
| Do not know or do not answer | 6 | 0.7 |
| Use of licit or illicit substance in the last three months |  |  |
| Yes | 380 | 46.8 |
| No | 422 | 52.0 |
| Do not know or do not answer | 10 | 1.2 |
| Substance used |  |  |
| Poppers | 47 | 5.8 |
| Cocaine | 55 | 6.8 |
| Marijuana | 197 | 24.3 |
| Club drugs | 12 | 1.5 |
| Erectile stimulants | 14 | 1.7 |
| Psychoactive drugs | 18 | 2.2 |
| Other | 15 | 1.8 |
| Do not know or do not answer | 454 | 55.9 |

**Table 1 (continuation)**

|  | n | % |
| --- | --- | --- |
| Frequency of condom use in anal sex in the last three months |  |  |
| None or less than half of the times | 219 | 26.9 |
| Half of the times | 76 | 9.4 |
| More than half or all the times | 387 | 47.6 |
| Did not have sex | 117 | 14.4 |
| Do not know or do not answer | 13 | 1.6 |
| Sex in the last three months |  |  |
| Receptive anal | 136 | 16.7 |
| Anal insertive | 327 | 40.3 |
| All sex with condom | 195 | 24.1 |
| Do not know or do not answer | 154 | 18.9 |
| **Health issues** | | |
| Lifelong diagnosis of syphilis |  |  |
| No | 125 | 15.4 |
| Yes | 685 | 84.4 |
| Do not know or do not answer | 2 | 0.2 |
| Positive syphilis test throughout life |  |  |
| Yes | 278 | 34.2 |
| No | 407 | 50.1 |
| Do not know or do not answer | 127 | 15.6 |
| Diagnosis of STIs throughout life |  |  |
| Yes | 442 | 54.4 |
| No | 352 | 43.3 |
| Do not know or do not answer | 18 | 2.2 |

**Table 1 (continuation)**

|  | n | % |
| --- | --- | --- |
| PrEP use |  |  |
| No | 665 | 81.9 |
| Yes | 129 | 15.9 |
| Do not know or do not answer | 18 | 2.2 |
| Start of PrEP use |  |  |
| One year ago or less | 62 | 7.6 |
| More than one year ago | 58 | 7.1 |
| Do not know or do not answer | 692 | 85.2 |
| Unprotected sex with people positive for syphilis in the last six months |  |  |
| Yes | 13 | 1.6 |
| No (I knew the person did not have syphilis infection) | 144 | 17.7 |
| I do not know if the person had syphilis infection | 350 | 43.1 |
| Do not know or do not answer | 305 | 37.6 |
| Consumption of five or more doses of alcohol within two hours in the last three months |  |  |
| Yes | 545 | 67.1 |
| No | 249 | 30.7 |
| Do not know or do not answer | 18 | 2.2 |
| Access to public health service |  |  |
| No | 34 | 4.2 |
| Yes | 769 | 94.7 |
| Do not know or do not answer | 9 | 1.1 |
| Last access to public health service |  |  |
| Over six months ago | 145 | 17.9 |
| Between two and six months ago | 227 | 28.0 |
| Within the last month | 350 | 43.1 |
| Do not know or do not answer | 90 | 11.1 |
| Access to FHS or Community Health Agent |  |  |
| No | 154 | 19.0 |
| Yes | 451 | 55.5 |
| Do not know/Do not answer | 207 | 25.5 |

**Table 1 (continuation)**

|  | n | % |
| --- | --- | --- |
| Supplemental health insurance |  |  |
| No | 364 | 44.8 |
| Yes | 439 | 54.1 |
| Do not know or do not answer | 9 | 1.1 |
| **App usage** | | |
| App as an environment where you met your partner |  |  |
| Yes | 501 | 61.7 |
| No | 211 | 26.0 |
| Do not know or do not answer | 100 | 12.3 |
| When began using the app |  |  |
| Since the beginning of the pandemic | 51 | 6.3 |
| Previous year | 120 | 14.8 |
| Since before the pandemic | 586 | 72.2 |
| Do not know or do not answer | 55 | 6.8 |
| Frequency of app use |  |  |
| Every day | 430 | 53.0 |
| A few days a week | 221 | 27..2 |
| Every day when receive a notification | 115 | 14.2 |
| Do not know or do not answer | 46 | 5.7 |
| Highest app usage period |  |  |
| All day | 361 | 44.5 |
| Morning, afternoon, or dawn | 114 | 14.0 |
| Night | 241 | 29.7 |
| Do not know or do not answer | 96 | 11.8 |
| Period of the week when most use the app |  |  |
| Weekdays | 423 | 52.1 |
| Weekends | 297 | 36.6 |
| Do not know or do not answer | 92 | 11.3 |
| Most used app in the last three months |  |  |
| Grindr^®^ | 482 | 59.4 |
| Tinder^®^ | 206 | 25.4 |
| Other | 116 | 14.3 |
| Do not know or do not answer | 8 | 1.0 |
| Purpose of app use |  |  |
| Sex | 347 | 42.7 |
| Relationship | 191 | 23.5 |
| Friendship | 51 | 6.3 |
| Hobby | 175 | 21.6 |
| Other | 38 | 4.7 |
| Do not know or do not answer | 10 | 1.2 |

N, number of participants; MW, minimum wage; Club drugs, ketamine, ecstasy, LSD, GHB, bath salts; Erection Stimulants, Viagra, Sildenafil, Cialis, Helleva, Levitra; Psychoactive drugs, Amphetamines, Anticholinergics, Barbiturates, Benzodiazepines, Opiates.
