# Supplementary material for: Prevalence and factors associated with syphilis among men who have sex with men in Brazil
Source: Front Public Health. 2025 May 8;13:1465799. doi: 10.3389/fpubh.2025.1465799 (PMC12094950; doi:10.3389/fpubh.2025.1465799)
Supplement: Supplementary file 2 [file Table_2.docx]

**Supplementary Table 2.** **Multivariate logistic regression of factors associated with lifelong diagnosis of syphilis among Brazilian MSM (*N* = 812).**

|  | | Lifelong diagnosis of syphilis | | | | | | | | | | | |
| --- | --- | --- | --- | --- | --- | --- | --- | --- | --- | --- | --- | --- | --- |
|  |  | Bivariate analysis | | | | |  | Multivariate analysis | | | | | |
|  |  | (N = 812) | | | | |  | (N = 812) | | | | | |
|  |  | n | (%) | p | PR | 95%CI |  | n | (%) | p | PR | aPR | a95%CI |
| Gender identity | Cis man | 227 | 30.9 | 0.044 | 2.16 | 0.75 - 6.20 |  |  |  |  |  |  |  |
|  | Transgender man | 8 | 53.3 |  | 3.73 | 1.18 - 11.79 |  |  |  |  |  |  |  |
|  | Non-binary | 3 | 14.3 |  | 1.00 | - |  |  |  |  |  |  |  |
| Sexual Orientation | Homosexual or gay | 208 | 32.5 | 0.117 | 1.27 | 0.93 - 1.73 |  |  |  |  |  |  |  |
|  | Bisexual | 34 | 25.6 |  |  |  |  |  |  |  |  |  |  |
| Skin color | White | 130 | 31.5 | 0.733 | 1.03 | 0.83 - 1.28 |  |  |  |  |  |  |  |
|  | Black | 108 | 30.3 |  |  |  |  |  |  |  |  |  |  |
| Age group | <30 years old | 108 | 24.1 | 0.000 | 0.60 | 0.48 - 0.74 |  | 108 | 24.1 |  |  |  |  |
|  | ≥30 years old | 135 | 39.9 |  |  |  |  | 135 | 39.9 | 0.002 | 1.66 | 1.49 | 1.15 - 1.92 |
| Educational level | High school | 44 | 31.4 | 0.885 | 1.02 | 0.77 - 1.33 |  |  |  |  |  |  |  |
|  | Higher education and Post Graduation | 199 | 30.8 |  |  |  |  |  |  |  |  |  |  |
| Work | No | 51 | 26.8 | 0.158 | 0.83 | 0.64 - 1.08 |  |  |  |  |  |  |  |
|  | Yes | 193 | 31.3 |  |  |  |  |  |  |  |  |  |  |
| Occupation | Student | 40 | 25.0 | 0.070 | 1.00 | - |  |  |  |  |  |  |  |
|  | Employed freelance professional | 31 | 32.0 |  | 1.27 | 0.86 - 1.89 |  |  |  |  |  |  |  |
|  | Self-employed professional | 44 | 39.3 |  | 1.57 | 1.10 - 2.23 |  |  |  |  |  |  |  |
|  | Employee or office worker | 49 | 27.8 |  | 1.11 | 0.77 - 1.59 |  |  |  |  |  |  |  |
| Income | One MW or less | 25 | 24.8 | 0.316 | 1.00 | - |  |  |  |  |  |  |  |
|  | Between one and three MW | 79 | 29.8 |  | 1.20 | 0.81 - 1.77 |  |  |  |  |  |  |  |
|  | Between three and five MW | 59 | 33.3 |  | 1.34 | 0.90 - 2.00 |  |  |  |  |  |  |  |
|  | Five or more MW | 80 | 34.2 |  | 1.38 | 0.94 - 2.02 |  |  |  |  |  |  |  |
| Region of residence | North | 34 | 35.8 | 0.105 | 1.50 | 1.04 - 2.15 |  |  |  |  |  |  |  |
|  | Northeast | 51 | 23.8 |  | 1.00 | - |  |  |  |  |  |  |  |
|  | Midwest | 41 | 31.8 |  | 1.33 | 0.94 - 1.88 |  |  |  |  |  |  |  |
|  | Southeast | 74 | 34.7 |  | 1.45 | 1.07 - 1.97 |  |  |  |  |  |  |  |
|  | South | 44 | 31.9 |  | 1.33 | 0.95 - 1.88 |  |  |  |  |  |  |  |
| Marital status | Single | 214 | 29.8 | 0.024 | 0.69 | 0.51 - 0.93 |  |  |  |  |  |  |  |
|  | Not single | 30 | 42.9 |  |  |  |  |  |  |  |  |  |  |
| Religiosity | No | 123 | 32.2 | 0.397 | 1.09 | 0.88 - 1.35 |  |  |  |  |  |  |  |
|  | Yes | 111 | 29.4 |  |  |  |  |  |  |  |  |  |  |
| Whom resides with | Alone | 92 | 31.4 | 0.992 | 1.05 | 0.76 - 1.44 |  |  |  |  |  |  |  |
|  | With parents | 73 | 30.9 |  | 1.03 | 0.74 - 1.44 |  |  |  |  |  |  |  |
|  | With relatives | 37 | 29.8 |  | 1.00 | - |  |  |  |  |  |  |  |
|  | With a colleague, friend, or partner | 42 | 31.1 |  | 1.04 | 0.72 - 1.50 |  |  |  |  |  |  |  |
| Impact of social distancing on sex life | High or medium impact | 174 | 31.9 | 0.423 | 1.09 | 0.87 - 1.38 |  |  |  |  |  |  |  |
|  | Low or none | 69 | 29.0 |  |  |  |  |  |  |  |  |  |  |

**Table 2** (continuation)

|  | | Lifelong diagnosis of syphilis | | | | | | | | | | | |
| --- | --- | --- | --- | --- | --- | --- | --- | --- | --- | --- | --- | --- | --- |
|  |  | Bivariate analysis | | | | |  | Multivariate analysis | | | | | |
|  |  | (N = 812) | | | | |  | (N = 812) | | | | | |
|  |  | n | (%) | p | PR | 95%CI |  | n | (%) | p | PR | aPR | a95%CI |
| Sex in the last three months | Yes | 225 | 33.3 | 0.000 | 1.98 | 1.29 - 3.02 |  |  |  |  |  |  |  |
|  | No | 19 | 16.8 |  |  |  |  |  |  |  |  |  |  |
| Number of cis men who had sex with | > Three cis men | 147 | 43.0 | 0.000 | 1.91 | 1.50 - 2.44 |  | 147 | 43.0 | 0.030 | 2.04 | 1.40 | 1.03 -1.90 |
|  | < Three cis men | 67 | 22.4 |  |  |  |  | 67 | 22.4 |  |  |  |  |
| Oral sex | Yes | 232 | 32.8 | 0.001 | 2.18 | 1.28 - 3.72 |  |  |  |  |  |  |  |
|  | No | 12 | 15 |  |  |  |  |  |  |  |  |  |  |
| Number of oral sex with cis men in the last three months | < Three cis men | 71 | 19.8 | 0.000 | 0.49 | 0.38 - 0.62 |  |  |  |  |  |  |  |
|  | > Three cis men | 159 | 40.5 |  |  |  |  |  |  |  |  |  |  |
| Sex in the last three months | Receptive and insertive | 193 | 32.8 | 0.939 | 1.01 | 0.75 - 1.35 |  |  |  |  |  |  |  |
|  | Insertive or receptive | 37 | 32.5 |  |  |  |  |  |  |  |  |  |  |
| Frequency of condom use in oral sex | None | 185 | 31.9 | 0.259 | 0.85 | 0.66 - 1.11 |  |  |  |  |  |  |  |
|  | Some or all of the time | 45 | 37.2 |  |  |  |  |  |  |  |  |  |  |
| Accepted money in exchange for sex | Yes | 64 | 43 | 0.000 | 1.53 | 1.22 - 1.91 |  |  |  |  |  |  |  |
|  | No | 179 | 28.1 |  |  |  |  |  |  |  |  |  |  |
| Paid for sex in the last three months | Yes | 12 | 37.5 | 0.545 | 0.86 | 0.52-1.41 |  |  |  |  |  |  |  |
|  | No | 50 | 43.5 |  |  |  |  |  |  |  |  |  |  |
| Sex worker | Yes | 05 | 41.7 | 0.871 | 0.94 | 0.47 - 1.89 |  |  |  |  |  |  |  |
|  | No | 56 | 44.1 |  |  |  |  |  |  |  |  |  |  |
| Chemosex practice | Yes | 96 | 39.5 | 0.001 | 1.45 | 1.18 - 1.79 |  |  |  |  |  |  |  |
|  | No | 146 | 27.1 |  |  |  |  |  |  |  |  |  |  |
| Chemosex practice in the last six months | < Three times | 48 | 31.8 | 0.002 | 0.60 | 0.43 - 0.82 |  |  |  |  |  |  |  |
|  | > Three times | 39 | 52.7 |  |  |  |  |  |  |  |  |  |  |
| Chemosex practice in the last two years | < Three times | 38 | 31.4 | 0.012 | 0.65 | 0.46 - 0.91 |  |  |  |  |  |  |  |
|  | > Three times | 48 | 48.0 |  |  |  |  |  |  |  |  |  |  |
| Sexual Positioning | Versatile | 158 | 35.1 | 0.047 | 1.44 | 1.05 - 1.96 |  |  |  |  |  |  |  |
|  | Insertive anal only | 36 | 24.3 |  | 1.00 | - |  |  |  |  |  |  |  |
|  | Receptive anal only | 47 | 30.7 |  | 1.26 | 0.87 - 1.82 |  |  |  |  |  |  |  |
| Casual sex during social distancing | Yes | 219 | 33.0 | 0.005 | 1.62 | 1.12 - 2.34 |  |  |  |  |  |  |  |
|  | No | 25 | 20.3 |  |  |  |  |  |  |  |  |  |  |
| Sex with two people or more simultaneously during social distancing | Yes | 143 | 40.5 | 0.000 | 1.76 | 1.42 - 2.19 |  | 143 | 40.5 | 0.053 | 1.77 | 1.31 | 1.00 - 1.73 |
|  | No | 99 | 22.9 |  |  |  |  | 99 | 22.9 |  |  |  |  |
| Use of licit or illicit substance in the last three months | Yes | 131 | 35.2 | 0.010 | 1.31 | 1.06 - 1.62 |  | 131 | 35.2 | 0.019 | 1.32 | 1.35 | 1.05 - 1.74 |
|  | No | 109 | 26.7 |  |  |  |  | 109 | 26.7 |  |  |  |  |

**Table 2** (continuation)

|  | | Lifelong diagnosis of syphilis | | | | | | | | | | | |
| --- | --- | --- | --- | --- | --- | --- | --- | --- | --- | --- | --- | --- | --- |
|  |  | Bivariate analysis | | | | |  | Multivariate analysis | | | | | |
|  |  | (N = 812) | | | | |  | (N = 812) | | | | | |
|  |  | n | (%) | p | PR | 95%CI |  | n | (%) | p | PR | aPR | a95%CI |
| Substance used | Poppers | 27 | 58.7 | 0.003 | 2.34 | 0.85 - 6.44 |  |  |  |  |  |  |  |
|  | Cocaine | 23 | 44.2 |  | 1.76 | 0.63 - 4.93 |  |  |  |  |  |  |  |
|  | Marijuana | 54 | 27.7 |  | 1.10 | 0.40 - 3.02 |  |  |  |  |  |  |  |
|  | Club drugs | 03 | 25.0 |  | 1.00 | - |  |  |  |  |  |  |  |
|  | Erectile or psychoactive stimulants | 13 | 39.3 |  | 1.57 | 0.54 - 4.58 |  |  |  |  |  |  |  |
|  | Other | 04 | 30.8 |  | 1.23 | 0.34 - 4.40 |  |  |  |  |  |  |  |
| Frequency of condom use in anal sex in the last three months | None | 33 | 36.3 | 0.002 | 1.67 | 1.15 - 2.42 |  | 33 | 36.3 |  |  |  |  |
|  | Less than half of the times | 60 | 50.0 |  | 2.30 | 1.68 - 3.14 |  | 60 | 50.0 | 0.016 | 1.69 | 1.42 | 1.07 - 1.89 |
|  | Half of the times | 32 | 42.1 |  | 1.94 | 1.34 - 2.80 |  | 32 | 42.1 |  |  |  |  |
|  | More than half of the times | 51 | 30.4 |  | 1.39 | 0.99 - 1.97 |  | 51 | 30.4 |  |  |  |  |
|  | Every time | 46 | 21.7 |  | 1.00 | - |  | 46 | 21.7 |  |  |  |  |
| Sex in the last three months | Receptive anal | 45 | 34.1 | 0.240 | 0.85 | 0.64 - 1.11 |  |  |  |  |  |  |  |
|  | Insertive anal | 128 | 40.0 |  |  |  |  |  |  |  |  |  |  |
| Syphilis testing throughout life | No | 07 | 6.0 | 0.000 | 0.17 | 0.08 - 0.35 |  |  |  |  |  |  |  |
|  | Yes | 236 | 35.2 |  |  |  |  |  |  |  |  |  |  |
| Positive test for syphilis | Yes | 206 | 75.7 | 0.000 | 10.1 | 7.11 - 14.34 |  |  |  |  |  |  |  |
|  | No | 30 | 7.5 |  |  |  |  |  |  |  |  |  |  |
| Diagnosis of STIs throughout life | Yes | 242 | 55.4 | 0.000 | 97.46 | 24.41 - 389.1 |  |  |  |  |  |  |  |
|  | No | 02 | 0.6 |  |  |  |  |  |  |  |  |  |  |
| PrEP use | No | 186 | 28.7 | 0.003 | 6.797 | 5.22 - 8.84 |  | 186 | 28.7 |  |  |  |  |
|  | Yes | 54 | 41.9 |  |  |  |  | 54 | 41.9 | 0.540 | 1.46 | 1.10 | 0.81 - 1.51 |
| Start of PrEP use | One year or less | 22 | 35.5 | 0.155 | 1.360 | 0.88 - 2.08 |  |  |  |  |  |  |  |
|  | More than one year | 28 | 48.3 |  |  |  |  |  |  |  |  |  |  |
| Unprotected sex with people positive for syphilis in the last six months | Yes |  |  | 0.005 |  |  |  |  |  |  |  |  |  |
|  | No (I knew the person did not have syphilis infection) | 5 | 38.5 |  | 1.5 | 0.71 - 3.16 |  |  |  |  |  |  |  |
|  | Do not know if the person had a syphilis infection | 141 | 41.2 |  | 1.61 | 1.18 - 2.19 |  |  |  |  |  |  |  |
| Alcohol consumption (≥ five doses) within two hours in the last three months | Yes | 174 | 32.6 | 0.103 | 1.21 | 0.95 - 1.55 |  |  |  |  |  |  |  |
|  | No | 64 | 26.8 |  |  |  |  |  |  |  |  |  |  |
| Access to public health service | No | 06 | 18.2 | 0.106 | 0.57 | 0.27 - 1.20 |  |  |  |  |  |  |  |
|  | Yes | 236 | 31.5 |  |  |  |  |  |  |  |  |  |  |

**Table 2** (continuation)

|  | | Lifelong diagnosis of syphilis | | | | | | | | | | | |
| --- | --- | --- | --- | --- | --- | --- | --- | --- | --- | --- | --- | --- | --- |
|  |  | Bivariate analysis | | | | |  | Multivariate analysis | | | | | |
|  |  | (N = 812) | | | | |  | (N = 812) | | | | | |
|  |  | n | (%) | p | PR | 95%CI |  | n | (%) | p | PR | PRaj | a95%CI |
| Last access to public health service | Over six months ago | 34 | 24.3 | 0.044 | 1.00 | - |  |  |  |  |  |  |  |
|  | Between two and six months ago | 72 | 32.6 |  | 1.34 | 0.94 - 1.90 |  |  |  |  |  |  |  |
|  | Within the last month | 124 | 36.0 |  |  |  |  |  |  |  |  |  |  |
| Access to FHS/Community Health Agent | No | 48 | 32.7 | 0.947 | 1.00 | 0.77 - 1.31 |  |  |  |  |  |  |  |
|  | Yes | 144 | 32.4 |  |  |  |  |  |  |  |  |  |  |
| Supplemental health insurance | No | 107 | 30.8 | 0.954 | 1.00 | 0.81 - 1.24 |  |  |  |  |  |  |  |
|  | Yes | 133 | 30.6 |  |  |  |  |  |  |  |  |  |  |
| App as an environment where they met the partner | Yes | 150 | 30.8 | 0.913 | 0.98 | 0.77 - 1.25 |  |  |  |  |  |  |  |
|  | No | 64 | 31.2 |  |  |  |  |  |  |  |  |  |  |
| When they began using the app | Since the beginning of the pandemic | 10 | 20.4 | 0.117 | 0.73 | 0.39 - 1.38 |  |  |  |  |  |  |  |
|  | Previous year | 32 | 27.6 |  | 1.00 | - |  |  |  |  |  |  |  |
|  | Since before the pandemic | 189 | 33.1 |  | 1.20 | 0.87 - 1.64 |  |  |  |  |  |  |  |
| Frequency of app use | Every day | 140 | 33.4 | 0.361 | 1.17 | 0.91 - 1.50 |  |  |  |  |  |  |  |
|  | A few days a week | 61 | 28.5 |  | 1.00 | - |  |  |  |  |  |  |  |
|  | Every day when receiving a notification | 32 | 28.6 |  | 1.00 | 0.69 - 1.43 |  |  |  |  |  |  |  |
| Highest app usage period | All day | 114 | 32.4 | 0.781 | 1.09 | 0.85 - 1.39 |  |  |  |  |  |  |  |
|  | Morning, afternoon, or dawn | 34 | 30.9 |  | 1.04 | 0.74 - 1.46 |  |  |  |  |  |  |  |
|  | Night | 70 | 29.7 |  | 1.00 | - |  |  |  |  |  |  |  |
| Week period when most use the app | During the weekdays | 135 | 32.8 | 0.111 | 1.20 | 0.95-1.52 |  |  |  |  |  |  |  |
|  | Weekends | 79 | 27.1 |  |  |  |  |  |  |  |  |  |  |
| Most used app in the last three months | Grindr^®^ | 173 | 37 | 0.000 | 2.04 | 1.48 - 2.80 |  | 173 | 37 | 0.028 | 1.63 | 1.38 | 1.04 - 1.83 |
|  | Tinder^®^ | 36 | 18.0 |  | 1.00 | - |  | 36 | 18.0 |  |  |  |  |
|  | Other | 34 | 30.9 |  | 1.71 | 1.14 - 2.57 |  | 34 | 30.9 |  |  |  |  |
| Purpose of app use | Sex | 123 | 36.2 | 0.062 | 1.42 | 1.07 - 1.89 |  |  |  |  |  |  |  |
|  | Relationship | 47 | 25.4 |  | 1.00 | - |  |  |  |  |  |  |  |
|  | Friendship | 14 | 29.8 |  | 1.17 | 0.70 - 1.94 |  |  |  |  |  |  |  |
|  | Hobby | 49 | 28.7 |  | 1.12 | 0.80 - 1.58 |  |  |  |  |  |  |  |

N, number of participants; PR, prevalence ratio; 95%CI, 95% confidence interval; aPR, adjusted prevalence ratio; a95%CI, adjusted confidence interval; MW, minimum wage; club drugs, ketamine, ecstasy, LSD, GHB, bath salts; Erection Stimulants, Viagra, Sildenafil, Cialis, Helleva, Levitra; psychoactive drugs, Amphetamines, Anticholinergics, Barbiturates, Benzodiazepines, and Opioids; FHS, Family Health Strategy.
